# Artichoke Polyphenols Rich in 1,4‐Dicaffeoylquinic Acid Regulate Lipid Metabolism via the PPAR and AMPK Pathways to Alleviate Hepatic Lipid Accumulation

**DOI:** 10.1002/cbdv.71750

**Published:** 2026-10-15

**Authors:** Wenyang Tao, Chenchen Yang, Quanqin Tan, Wanyi Zhou, Jianrong Xing, Jingrui Li, Mengfan Luo, Ying Yang

**Affiliations:** ^1^ Institute of Food Science Zhejiang Academy of Agricultural Sciences Hangzhou Zhejiang China

**Keywords:** AMPK, Artichoke polyphenols, HPLC‐MS, lipid‐lowering, Obesity, PPAR

## Abstract

Given the global surge in obesity, this study evaluated artichoke polyphenols (APs) as potential functional agents against obesity‐related hepatic lipid accumulation by elucidating their chemical composition and underlying mechanisms. Semi‐quantitative HPLC‐MS analysis revealed that 1,4‐dicaffeoylquinic acid and chlorogenic acid constituted 60–80% of the total polyphenolic peak areas, with inner bract polyphenols (IBAPs) exhibiting higher purity and phenolic acid content than outer bract polyphenols (OBAPs). In an oleic acid‐induced high‐fat HepG2 cell model, APs at non‐cytotoxic concentrations dose‐dependently and significantly reduced intracellular lipid droplet accumulation, triglyceride, and total cholesterol levels. Mechanistically, APs were correlated with activation of the AMPK signaling pathway, markedly upregulating AMPKα, phosphorylated AMPKα, and CPT1A, while concurrently downregulating FABP4, and PPARγ. These findings suggest that artichoke bract polyphenols are associated with alleviation of hepatic lipid accumulation, potentially through coordinated modulation of PPAR and AMPK pathways that correlate with enhanced mitochondrial fatty acid oxidation and reduced de novo lipogenesis, highlighting their potential as natural functional agents that warrant further investigation for managing obesity‐related metabolic disorders.

## Graphical abstract

Artichoke bract polyphenols, particularly from inner bracts, are rich in 1,4‐dicaffeoylquinic acid and chlorogenic acid. In oleic acid‐induced HepG2 cells, these polyphenols activate AMPK and modulate PPARγ signaling, reducing lipid accumulation. These findings highlight the potential of artichoke bracts, including agricultural waste outer bracts, as functional ingredients for managing obesity‐related metabolic disorders.

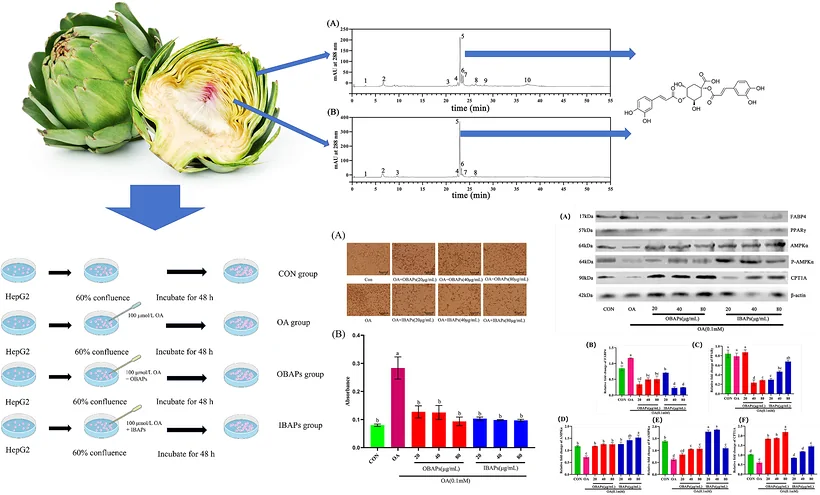

## 1 Introduction

Artichoke (*Cynara scolymus L*.) has been revered for over two thousand years for its medicinal and culinary uses [1]. Its significant edible and functional properties have made it a staple in various cuisines aimed at disease prevention and bodily function regulation. The key bioactive compounds in artichokes, including polysaccharides, alkaloids, and polyphenols, contribute to these beneficial effects [2, 3]. The metabolic of plant processes give rise to specific secondary metabolites, namely polyphenolic compounds, due to the phenolic hydroxyl groups in their molecular structures [4]. These polyphenolic compounds are categorized into monomers and polymers, with monomers further divided into flavonoids and phenolic acids, and polymers consisting of hydrolyzable and condensed tannins [5]. The antioxidant properties of polyphenols, attributed to their ability to donate electrons to peroxidizing free radicals, highlight the plant's functional potential [6].

In recent clinical studies, artichoke leaf extracts have been shown to decrease cholesterol synthesis in the liver and lower blood cholesterol levels, thereby reducing fat accumulation in other tissues [7]. These findings support the use of artichoke as a choleretic and hepatoprotective agent, beneficial for treating liver function abnormalities and dyspepsia [8]. Moreover, the ability of extracts to protect proteins, lipids, and DNA from oxidative damage underscores their significant antioxidant and antifungal activities [9]. The focus of current chemical research on artichoke extracts includes the study of phenolic acids (caffeoylquinic acids), luteolin, and apigenin, along with their glycoside compounds, indicating a broad spectrum of bioactive compounds contributing to these effects [10, 11].

As widely acknowledged, obesity results from the intricate interplay of environmental and genetic factors [12]. Recent statistics reveal a concerning rise in the number of obese individuals, underscoring the imperative for global collaboration in tackling the reduction of obesity. Obesity and its associated metabolic syndrome components such as insulin resistance, type 2 diabetes (T2D), nonalcoholic fatty liver disease (NAFLD), inflammation, hypertension, and atherosclerosis pose significant health risks [13, 14]. The fundamental cause of obesity, an imbalance between energy intake and expenditure leading to excessive fat accumulation, underscores the importance of identifying effective preventive and intervention strategies [15]. In this context, the role of peroxisome proliferator‐activated receptor gamma (PPARγ) in adipocyte differentiation and lipid storage and mobilization [16, 17]. Along with the activation of lipid metabolism‐regulating signaling pathways like AMP‐activated protein kinase (AMPK), it becomes particularly relevant [18].

The established benefits of artichoke in lipid metabolism and its antioxidant properties suggest a promising avenue for obesity management. However, despite clear evidence on the composition of polyphenolic compounds in artichokes, the detailed study of polyphenols across different artichoke varieties and plant parts, and their specific impacts on obesity and metabolic syndrome, remains an area ripe for further research. This gap highlights the need for a more systematic exploration of artichoke's bioactive compounds and their mechanisms of action in combating obesity‐related conditions. Hence, this paper focuses on utilizing the bracts of artichoke flower heads as raw materials to extract polyphenols, identify their chemical composition, and conduct cell experiments to explore their potential in modulating lipid metabolism initially.

Artichoke bracts are anatomically and functionally distinct. The outer bracts are tough, green, and exposed to environmental stress, whereas the inner bracts are tender, pale, and protected, suggesting potentially different phytochemical accumulation patterns. From a practical perspective, outer bracts are predominantly discarded as agricultural waste during artichoke processing, while inner bracts are consumed as the edible portion. Thus, comparing their polyphenolic profiles and biological activities may inform whether the discarded outer bracts retain sufficient bioactivity to serve as a low‐cost source of functional ingredients. Our preliminary data further indicated that the two bract types differ substantially in phenolic acid content, providing an opportunity to explore whether such compositional differences translate into differential lipid‐lowering efficacy.

## 2 Materials and Methods

### 2.1 Materials and Reagent

Artichokes (*Cynara scolymus* L.) were purchased from a local agricultural market in Zhejiang, China, in September 2024. The plant material was commercially cultivated and identified by the supplier based on its horticultural variety. Representative samples of the raw materials have been retained in our laboratory for future reference. As the material was commercially purchased fresh produce rather than wild‐collected, a herbarium voucher specimen was not deposited. Oleic acid (OA, ≥99%, Cell Culture Grade), Modified Oil Red O Staining Kit, Cell Counting Kit‐8 (CCK‐8), BCA protein assay kit, Cell lysis buffer for Western and IP, Protease and phosphatase inhibitor cocktail for general use, polyvinylidene difluoride filters membrane (PVDF), 4% Paraformaldehyde (PFA) Fix Solution, and dimethyl sulfoxide (DMSO) (≥99.9%, BioReagent) were purchased from Shanghai Beyotime Biotechnology Co., Ltd (Shanghai, China). The Total Cholesterol (TC) Assay Kit and Triglycerides (TG) Assay Kit were purchased from Nanjing Jiancheng Bioengineering Institute (Nanjing, China). HepG2 cells (RRID: CVCL_0027) and HepG2 cell‐specific medium (90%MEM + 10%FBS + 1%P/S) were purchased from Wuhan Boster Biological Technology Co., Ltd (Wuhan, China). TBST buffer, primary antibodies (AMPKα, P‐AMPKα, FABP4, PPARγ, CPT1A), sheep anti‐rabbit IgG‐HRP, and chemiluminescence Efficient chemiluminescence (ECL) kit were purchased from Proteintech Group, Inc. (Chicago, USA).

### 2.2 APs Extract Preparation

The outer and inner bracts of artichokes were separated, freeze‐dried, and ground into a fine powder (passed through a 40‐mesh sieve). Polyphenols were extracted using a 40% ethanol‐water solution (v/v) with a solid‐liquid ratio of 1:30 (w/v) at 60°C for 2 h under constant stirring (200 rpm). The extraction was repeated three times, and the combined filtrates were filtered through a 200‐mesh nylon cloth. The pooled filtrate was concentrated under reduced pressure at 50°C. The concentrated extract was then purified using D101 macroporous adsorption resin (20 cm × 4.5 cm i.d., 300 mL bed volume). The resin was preconditioned according to the manufacturer's instructions. After sample loading, the column was washed with distilled water (3 bed volumes) to remove polar impurities, and the polyphenols were eluted with a 70% ethanol‐water solution (v/v, 3 bed volumes) at a flow rate of 2.0 mL/min. The eluate was collected, concentrated, and freeze‐dried to obtain the artichoke polyphenol extract. The extraction yields (calculated as extract weight / dry material weight × 100%) were 5.802 ± 0.488% for OBAPs and 14.314 ± 0.182% for IBAPs (mean ± SD, *n* = 3). The purities of the final extracts, determined by the Folin‐Ciocalteu method and expressed as chlorogenic acid equivalents, were 75.62 ± 2.45% for OBAPs and 82.57 ± 3.78% for IBAPs. Purification recovery rates were not determined in this study. The purities reported above represent the total phenolic content determined by the Folin‐Ciocalteu method, expressed as chlorogenic acid equivalents. This method is specific to phenolic compounds but does not exclude the presence of other nonphenolic constituents such as residual sugars, proteins, or other polar compounds that may have coextracted and copurified during the process. The remaining mass of the extracts (approximately 17%–24%) likely includes such non‐phenolic components, although their precise composition was not determined in this study. The lyophilized APs extracts were stored as dry powders at −20°C in amber vials protected from light and moisture until further use.

### 2.3 HPLC and Mass Spectrometric Identification of APs

Dissolve the samples in methanol and pass it through 0.22 µM membrane filter filters supernatant. After that, Samples were analyzed using an Agilent Technologies 1200 series HPLC (Agilent Technologies, Santa Clara, CA, USA) equipped with a diode array detector and a ZORBAX Eclipse XDB‐C18 (4.6 x 150 mm, 5 um, Agilent Technologies, CA, USA). Then 0.1% formic acid aqueous solution as solvent A, and Acetonitrile was employed as solvent B in the mobile phase. Using a flow rate of 0.5 mL/min, the gradient elution was as follows: 0 min 8% B, 5 min 8% B, 10 min 20% B, 18 min 20% B, 22 min 30% B, 34 min 40%, 40 min 85% B, 50 min 85% B, and 55 min 8% B. The detection was recorded at 288 nm, and the column oven was adjusted to 35°C. Using a Q‐TOF 6550 (Agilent Technologies, Santa Clara, CA, USA) equipment in negative mode with *m/z* values ranging from 100 to 1200, a mass spectrometric study was performed. The following were the other settings for mass spectrometry (MS): 350°C for the nitrogen gas; 12 L/min for the drying gas; 40 psi for the nebulization pressure; 15 V for the cone voltage; and 3.2 kV for the capillary voltage.

### 2.4 Cell Culture

HepG2 cells were cultured in HepG2 cell‐specific cultivators solution with 10% FBS, 50 lg/mL of streptomycin and 50 units/mL of penicillin. The cells were maintained in an incubator with 5% CO_2_ at 37°C. After reaching 60% confluence, the cells were treated with 100 µmol/L OA, and with APs at different concentrations for 48 h. The concentration of OA (100 µmol/L) was selected based on preliminary experiments showing that it induced a 1.8‐fold increase in intracellular TG content compared with the control group, while maintaining cell viability above 90% as determined by CCK‐8 assay (Section 2.5). The APs concentrations (20, 40, and 80 µg/mL) were selected based on the CCK‐8 cytotoxicity results (Figure 3), which confirmed that APs at concentrations up to 120 µg/mL did not affect cell viability. These three concentrations within the safe range were chosen to evaluate potential dose‐dependent effects of APs on lipid metabolism. For each experiment, the APs extracts were freshly dissolved in culture medium immediately before treatment to minimize potential degradation. The detailed process of cell culture is shown in Figure 1.

**FIGURE 1:**
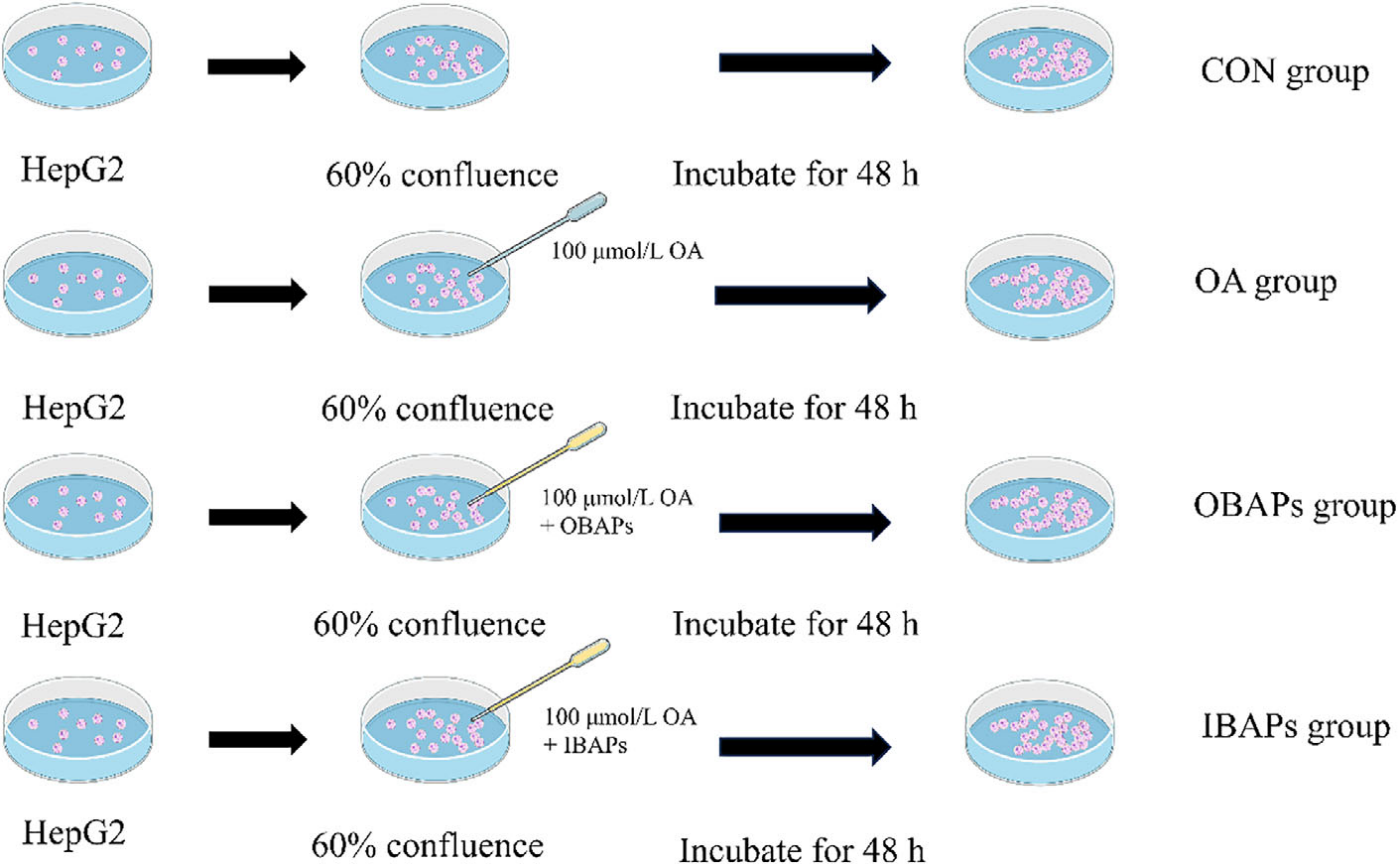
HepG2 cell experiment arrangement. OBAPs and IBAPs groups were set concentration gradients of 20, 40, and 80 µg/mL.

### 2.5 Cell Viability

The cytotoxicity of APs was assessed by the Cell Counting Kit‐8 (CCK‐8). HepG2 cells were seeded at a density of 5.0 × 10^3^ cells/well in 96‐well plates and cultured to reach 60% confluence. Then, cells were treated with different concentrations of APs (20, 40, and 80 µg/mL) for 48 h. After that, 10 µl of CCK‐8 solution was added to each well and cultured in a cell growth chamber for 1 h before measuring absorbance at 450 nm. Sample concentrations having absorbance readings less than 10% lower than the control were considered to be noncytotoxic to cells.

### 2.6 HepG2 Cell Staining With Oil Red O

Oil Red O staining was carried out using a Modified Oil Red O Staining Kit in accordance with the instructions of the manufacturer. After the end of cell culture, remove the medium, use PBS cleaning twice, and then add 4% polymorphide fixed for 30 min. Cells were treated with the Oil Red O solution for 15 min following two PBS washes. The unbound dye was then removed from the cells by rinsing them twice with PBS. A microscope (Olympus IX73, Olympus Corporation, Tokyo, Japan) with an inbuilt camera was used to capture images of labeled cells. Based on the Yu method [18], after being air‐dried for a whole night, the stained cells were dissolved in isopropanol, and the absorbance at 520 nm was measured. Image acquisition and quantification were performed using standardized procedures (fixed exposure time and consistent microscope settings for all samples). The investigators were aware of the treatment groups during image acquisition and quantification, and blinding was not formally implemented. All images were acquired using an Olympus IX73 microscope (Olympus Corporation, Tokyo, Japan) with consistent exposure settings, gain, and magnification across all samples. A scale bar of 50 µm was applied to all representative images.

### 2.7 Preparation of Protein Extract of HepG2 Cells

HepG2 cells were washed with PBS twice, and then cell lysis buffer Cell lysis buffer with Protease and phosphatase inhibitors was added to the cells on ice for 30 min. Then, the mixtures were centrifuged at 10,000 × g at 4°C for 20 min. The protein concentration of sample was determined by a BCA protein assay kit.

### 2.8 Cellular Cholesterol (TC) and Triacylglycerol (TG) Contents Analysis

HepG2 were seeded in 6‐well plates and treated under different conditions (CON; OA; cotreated with APs and OA). Then, as was previously indicated, cell lysates were made. The Total Cholesterol Assay Kit and the Triacylglycerol Assay Kit were used to measure the TC and TG contents, following the directions provided by the manufacturer. The protein concentration of each cell lysate sample was determined using the BCA protein assay kit (Section 2.7), and TC and TG contents were normalized to total cellular protein, expressed as mmol of lipid per gram of cellular protein (mmol/g protein).

### 2.9 Western Blot Assay

SDS‐polyacrylamide gel electrophoresis was used to separate equal amounts of protein (15–20 µg), which were subsequently transferred to a PVDF membrane. Pre‐stained molecular weight markers (PageRuler Prestained Protein Ladder, Thermo Scientific) were run alongside the samples to confirm protein size. The membrane was blocked for one hour at 4°C using 5% nonfat milk in TBST, and then incubated with the primary antibodies overnight at 4°C. The primary antibodies used were as follows: anti‐AMPKα (1:1000), anti‐phospho‐AMPKα (Thr172, 1:1000), anti‐FABP4 (1:1000), anti‐PPARγ (1:1000), anti‐CPT1A (1:1000), and anti‐*β*‐actin (1:5000, loading control). After washing with TBST, the membranes were incubated for 90 min at 25°C with goat anti‐rabbit IgG‐HRP (1:1000). Protein bands were visualized using a chemiluminescence ECL kit following the manufacturer's instructions, and images were captured on x‐ray film. Densitometric analysis of the target bands was performed using ImageJ software (NIH, Bethesda, MD, USA), with *β*‐actin as the internal control for normalization.

### 2.10 Statistical Analysis

All data are presented as mean ± standard error of the mean (SEM) from at least three independent biological replicates (*n* = 3). For each independent experiment, technical replicates were performed as follows: CCK‐8 assay (*n* = 6 technical replicates per condition), Oil Red O staining (*n* = 3 technical replicates), TG/TC quantification (*n* = 3 technical replicates), and Western blot analysis (*n* = 3 technical replicates). Normality of data distribution was assessed using the Shapiro–Wilk test, and homogeneity of variances was verified using Levene's test. For multi‐group comparisons, one‐way analysis of variance (ANOVA) was performed, followed by Tukey's multiple comparisons post‐hoc test to identify significant differences between individual groups. Statistical significance was set at *p < 0.05. All statistical analyses were performed using GraphPad Prism 8.0 (GraphPad Software, San Diego, CA, USA) and Microsoft Excel 2019.

## 3 Results

### 3.1 Identification of APs Composition by HPLC‐MS Spectrometry

The tentative identification of the major polyphenolic components in APs was performed by HPLC‐MS/MS analysis, and the results are delineated in Figure 2, with Figure 2A,B portraying the ultraviolet chromatograms of OBAPs and IBAPs, correspondingly. Ten suspected substance peaks were detected in the OBAPs and eight in the IBAPs, each designated with a response time at which the peak emerged. From Figure 2, it is evident that there are 7 overlapped peaks between the two APs, including peaks 1, 2, 4, 5, 6, 7, and 8 in Figure 2A and peaks 1, 2, 4, 5, 6, 7, and 8 in Figure 2B. Peaks 3, 9, and 10 in Figure 2A are unique to the OBAPs, whereas peak 3 in Figure 2B is exclusive to the IBAPs. Thus, there may be a total of seven substances shared between these two polyphenolic extracts, with OBAPs comprising three unique compounds and IBAPs containing only one.

**FIGURE 2:**
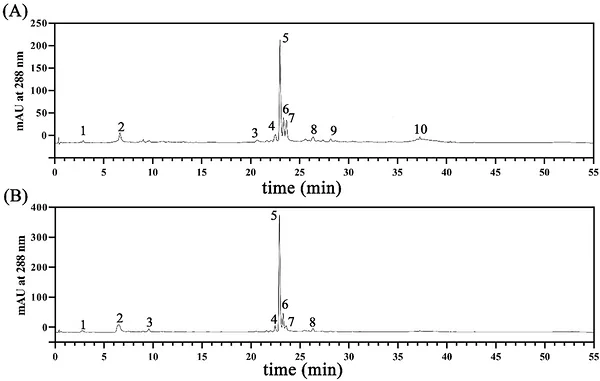
HPLC chromatograms of APs. (A) HPLC chromatograms of OBAPs, (B) HPLC chromatograms of IBAPs.

To further elucidate the precise chemical composition, following the work of Hu et al. [8], it is postulated that the compounds corresponding to chromatographic peaks labeled 2, 6, 7, and 8 in Figure 2A are chlorogenic acid (3‐caffeoylquinic acid), luteoloside, luteolin‐7‐O‐glucoside, and apigenin‐7‐O‐glucoside. Additionally, based on the findings of Abu‐Reidah et al. [19], the compounds corresponding to chromatographic peaks labeled 3 and 10 in Figure 2A are luteolin and apigenin. The compound denoted by peak 4 in Figure 2A is tentatively identified as (+)‐Pinoresinol‐O‐beta‐D‐glucopyranoside [20]. Lastly, a query in the MassBank database (https://massbank.eu/ MassBank/) uncovers that the compounds corresponding to chromatographic peaks 1, 5, and 9 in Figure 2A and peak 3 in Figure 2B are 3,4‐dihydroxybenzyl aldehyde, 1,4‐dicaffeoylquinic acid, dimethoxycinnamic acid, and 4‐coumaroylquinic acid.

In accordance with the preceding discourse, the identification results of OBAPs and IBAPs are shown in Tables 1 and 2, respectively. The relative content of 1,4‐dicaffeoylquinic acid is highest in both APs, followed by chlorogenic acid. Furthermore, the relative content of total flavonoids (luteolin, luteoloside, luteolin‐7‐O‐glucoside, apigenin‐7‐O‐glucoside, apigenin, and apigenin‐7‐O‐glucoside) was higher in OBAPs based on peak area percentages. In contrast, the relative content of phenolic acids (chlorogenic acid, 4‐coumaroylquinic acid, 1,4‐dicaffeoylquinic acid, and dimethoxycinnamic acid) is higher in IBAPs.

**TABLE 1:** Identification of OBAPS compositions.

Peak no.	RT time	Formula	MS[M‐H]‐	MS‐MS	Name	Relative abundance
1	3.514	C7H6O3	138.0317	135.0121; 109.0335	3,4‐Dihydroxybenzyl aldehyde	1.07%
2	6.506	C16H18O9	354.0952	135.756; 179.0267; 191.3426	Chlorogenic acid	7.61%
3	20.65	C15H1O6	287.0562	112.9833; 135.0439; 151.0032	Luteolin	2.70%
4	22.486	C26H32O11	520.1946	381.12; 441.175; 461.2378	(+)‐Pinoresinol‐O‐beta‐D‐gucopyranoside	5.78%
5	23.064	C25H24O12	515.1198	179.5813; 191.659; 353.9562	1,4‐Dicaffeoylquinic acid	56.08%
6	23.2	C21H20O11	447.0936	287.1451; 288.1029	Luteoloside	10.40%
7	23.778	C27H30O15	594.1512	269.1247; 271.376; 432.9267	Luteolin 7‐O‐rutinoside	9.23%
8	26.362	C27H30O14	577.1565	271.3762; 417.8541; 433.0247	Apigenin‐7‐O‐ rutinoside	4.16%
9	27.79	C11H12O4	208.0736	105.4301;117.0145	Dimethylcaffeic acid	1.93%
10	37.343	C15H10O5	270.0531	119.1247;153.0177	Apigenin	1.04%

*Note*: The relative abundance of each compound was calculated as the percentage of its peak area relative to the total peak area of all detected compounds in the HPLC chromatogram at 288 nm.

**TABLE 2:** Identification of IBAPS compositions.

Peak no.	RT time	Formula	MS[M‐H]‐	MS‐MS	Name	Relative abundance
1	3.449	C7H6O3	138.0317	135.0121; 109.0335	3,4‐Dihydroxybenzyl aldehyde	1.20%
2	6.61	C16H18O9	354.0952	135.756; 179.0267; 191.3426	Chlorogenic acid	12.07%
3	9.636	C16 H18O8	338.1004	163.0483; 191.0562	4‐Coumaroylquinic acid	2.47%
4	22.453	C26H32O11	520.1946	381.12; 441.1759; 461.2378	(+)‐Pinoresinol‐O‐beta‐D‐glucopyranoside	3.51%
5	22.963	C25H24O12	515.1198	179.5813; 191.659; 353.9562	1,4‐Dicaffeoylquinic acid	68.90%
6	23.337	C21H20O11	447.0936	287.1451; 288.1029	Luteoloside	7.27%
7	23.677	C27H30O15	594.1512	269.1247; 271.376; 432.9267	Luteolin 7‐O‐rutinoside	2.11%
8	26.397	C27H30O14	577.1565	271.3762; 417.8541; 433.0247	Apigenin‐7‐O‐ rutinoside	2.47%

*Note*: The relative abundance of each compound was calculated as the percentage of its peak area relative to the total peak area of all detected compounds in the HPLC chromatogram at 288 nm.

### 3.2 Effects of APs on HepG2 Cell Viability

To confirm that the intervention with APs did not have any negative impact on cellular viability, we conducted the CCK8 assay for cellular toxicity. As shown in Figure 3, we found that the viability of HepG2 cells exposed to OA remained unaffected when treated with 0–120 µg/mL of APs. However, treatment with 120–200 µg/mL of APs had an impact on the viability of these cells. Based on these findings, we selected 20, 40, and 80 µg/mL of APs for the next experiments.

**FIGURE 3:**
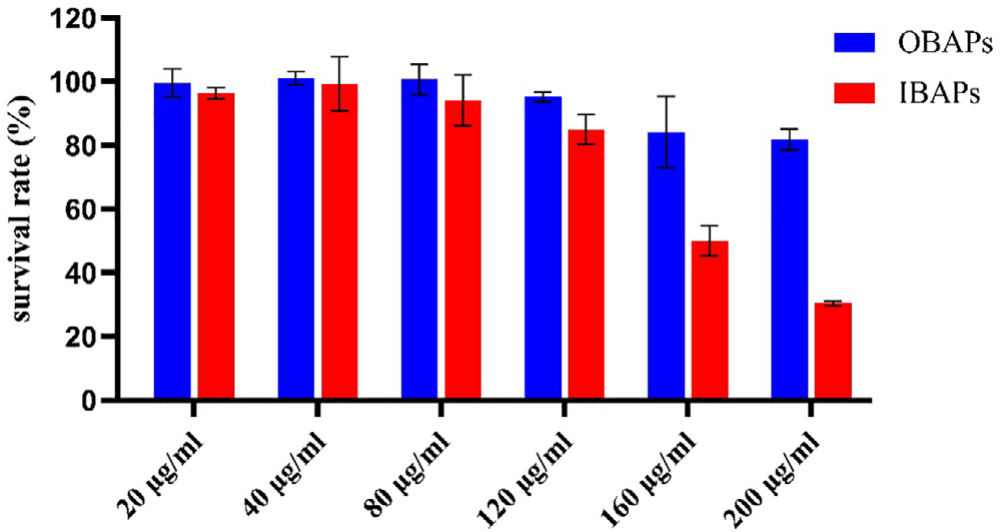
The cell viability after treatment with APs and OA. Data are presented as mean ± SEM from three independent biological replicates (*n* = 3).

### 3.3 Effects of APs on Lipid Accumulation in OA‐Treated HepG2 Cells

The image of Oil Red O‐stained cells is shown in Figure 4A. A comparison between the control group (CON) and the high‐fat group (OA) revealed a higher presence of red lipid droplets within the cells of the OA group, indicating increased lipid accumulation induced by OA. Subsequent Oil Red O staining of the OBAPs and IBAPs groups demonstrated a noticeable reduction in lipid accumulation within the cells post‐treatment. Moreover, as the polyphenol dosage increased, a proportional decrease in lipid accumulation within the cells was observed, with the cells treated with 80 µg/mL of OBAPs exhibiting the least internal lipid accumulation.

**FIGURE 4:**
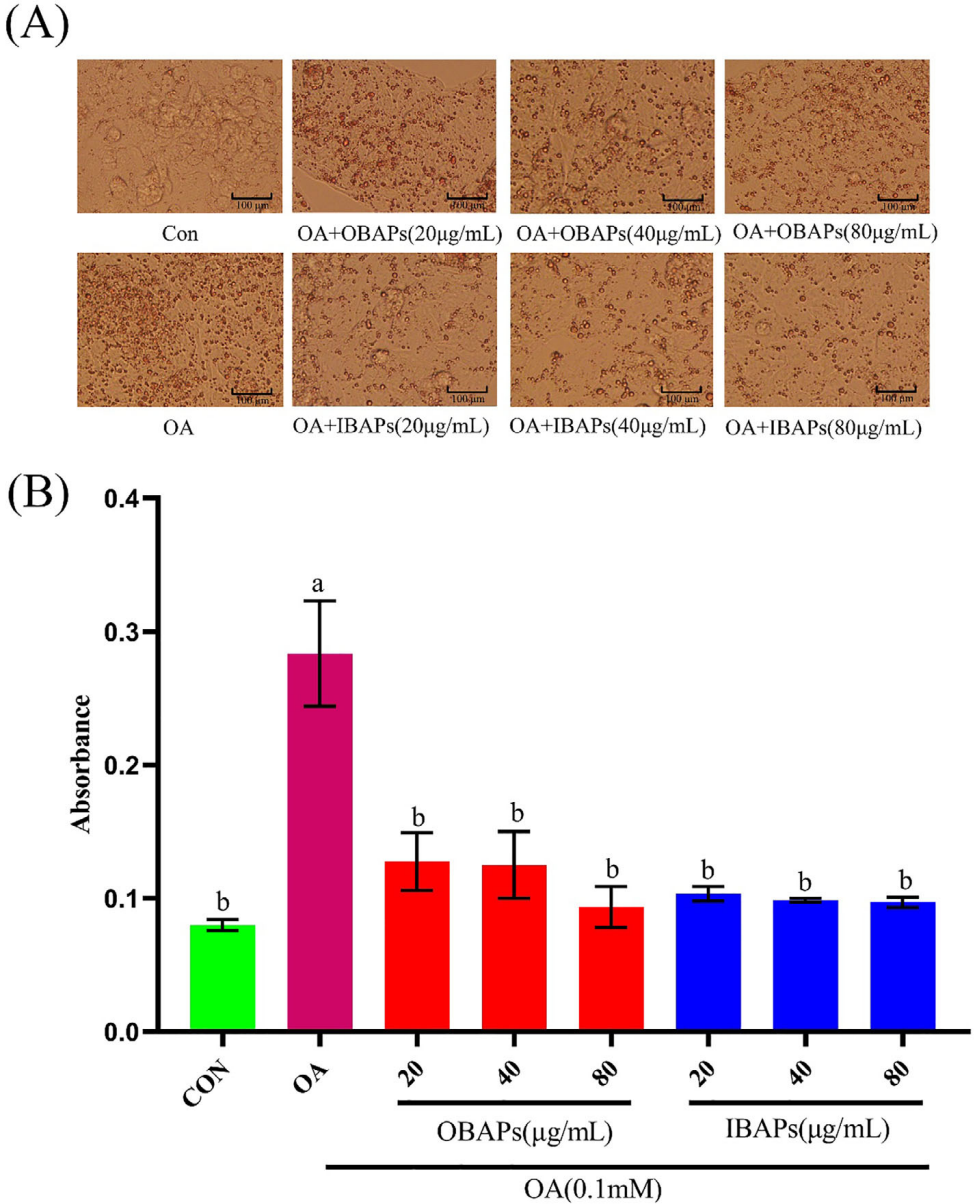
The results of Oil Red O staining. (A) The represented images of Oil Red O staining results; (B) the absorbance of oil droplets at 520 nm. Data were represented as mean ± SEM and analyzed by one‐way ANOVA with Tukey's post‐hoc test. Significant differences (*p* < 0.05) are indicated with different letters (a, b). Data are presented as mean ± SEM from three independent biological replicates (*n* = 3).

Furthermore, Figure 4B illustrates that after the isopropanol extraction of oil droplets in each cell group, the absorbance at 520 nm of the isopropanol extracts from the CON group, OBAPs group, and IBAPs group was significantly lower than that of the high‐fat group (*p* < 0.05). Additionally, there were no significant differences in absorbance between different concentrations of the OBAPs and IBAPs groups (*p* > 0.05), and the absorbance did not significantly differ from that of the control group (*p* > 0.05). Therefore, it can be concluded that APs effectively reduce lipid accumulation in HepG2 cells following OA treatment.

### 3.4 Effects of APs on TG and TC Contents in OA‐Treated HepG2 Cells

According to Figure 5, the content of total cholesterol (TC) and triglycerides (TG) in cells significantly increased after OA treatment compared to the control (CON) group (*p* < 0.05). However, treatment with APs notably attenuated the elevation of TC and TG induced by OA (*p* < 0.05), particularly with 80 µg/mL of OBAPs and IBAPs. Moreover, the TG content in cells treated with 80 µg/mL of OBAPs and 40 µg/mL and 80 µg/mL of IBAPs was significantly lower than that in the CON group (*p* < 0.05). Therefore, it can be concluded that APs effectively reduce TC and TG levels and ameliorate the abnormal lipid metabolism of HepG2 cells caused by OA treatment.

**FIGURE 5:**
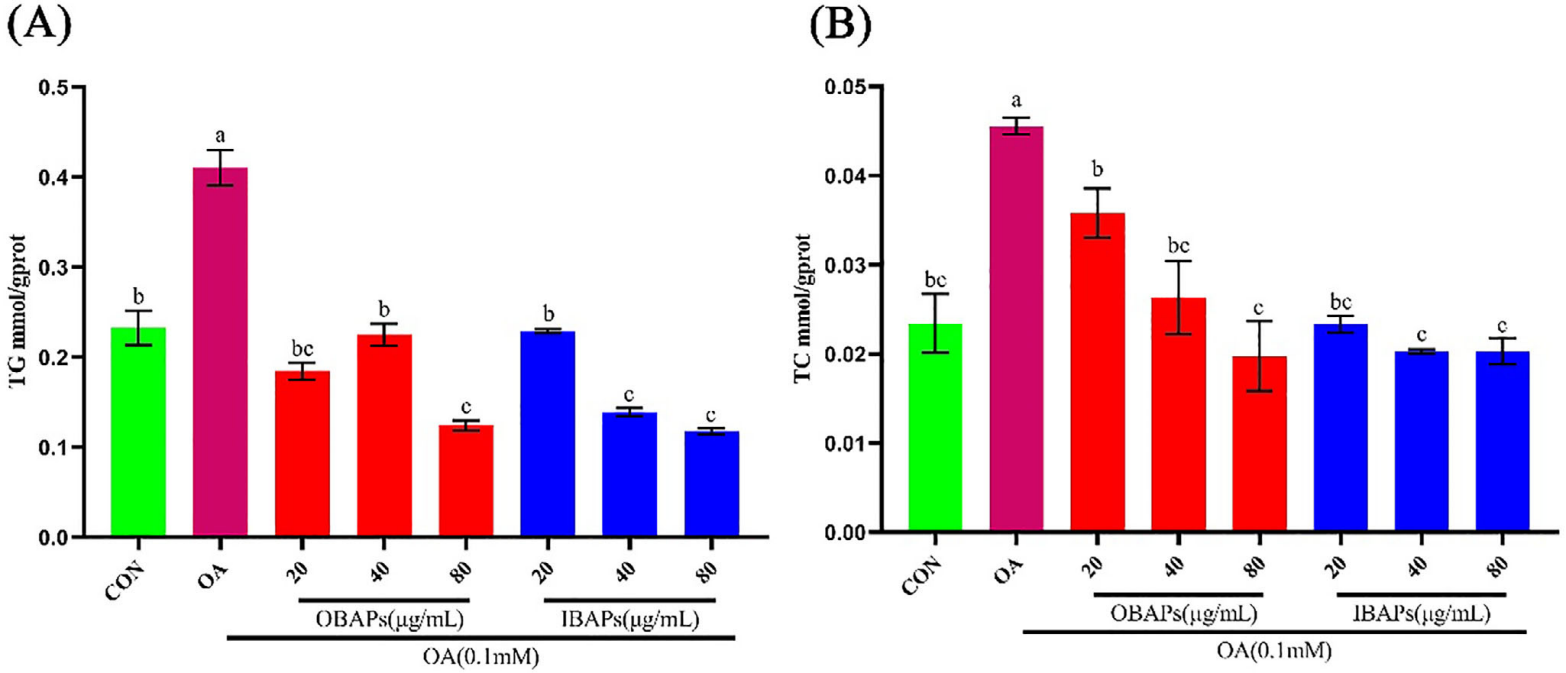
The results of TG and TC assessment. (A) The level of TG in HepG2 cells; (B) The level of TC in HepG2 cells. Data are represented as mean ± SEM and analyzed by one‐way ANOVA with Tukey's post‐hoc test. Significant differences (*p* < 0.05) are indicated with different letters (a–c). Data are presented as mean ± SEM from three independent biological replicates (*n* = 3).

### 3.5 Effects of APs on the PPAR and AMPK Signaling Pathways in HepG2 Cells

The results of the HepG2 cell Western blot experiment are depicted in Figure 6. In Figure 6A, the quantified protein expression levels determined through Western blot are illustrated. As observed in Figure 6B, compared to the control (CON) group, oleic acid (OA) induces an increase in the expression levels of FABP4 in HepG2 cells. However, supplementation with APs notably decreases the expression levels of FABP4, particularly with the addition of 40 and 80 µg/mL of IBAPs (*p* < 0.05). According to Figure 6C, supplementation with 20 µg/mL OBAPs does not significantly alter the expression levels of PPARγ in HepG2 cells (*p* > 0.05); however, other doses of APs, notably 40 µg/mL OBAPs, can significantly decrease the expression levels of PPARγ (*p* < 0.05). Additionally, an increase in the concentration of IBAPs leads to a gradual rise in the expression levels of PPARγ. Figure 6D,E show that OA treatment decreased both AMPKα and P‐AMPKα expression in HepG2 cells compared with the control group. Supplementation with APs significantly increased the expression of both proteins (p < 0.05). Notably, the increase in P‐AMPKα was proportionally greater than that of total AMPKα across all APs treatment groups, suggesting an elevated P‐AMPK/AMPKα ratio and indicating enhanced AMPK activation, with IBAPs exhibiting a more pronounced effect. In Figure 6F, it is revealed that OA can suppress the expression of CPT1A compared to the control group, while supplementation with OBAPs correlated with the expression of CPT1A. Furthermore, IBAPs can alleviate the OA‐induced downregulation of CPT1A expression, with an incremental concentration of IBAPs leading to a gradual increase in the expression levels of CPT1A.

**FIGURE 6:**
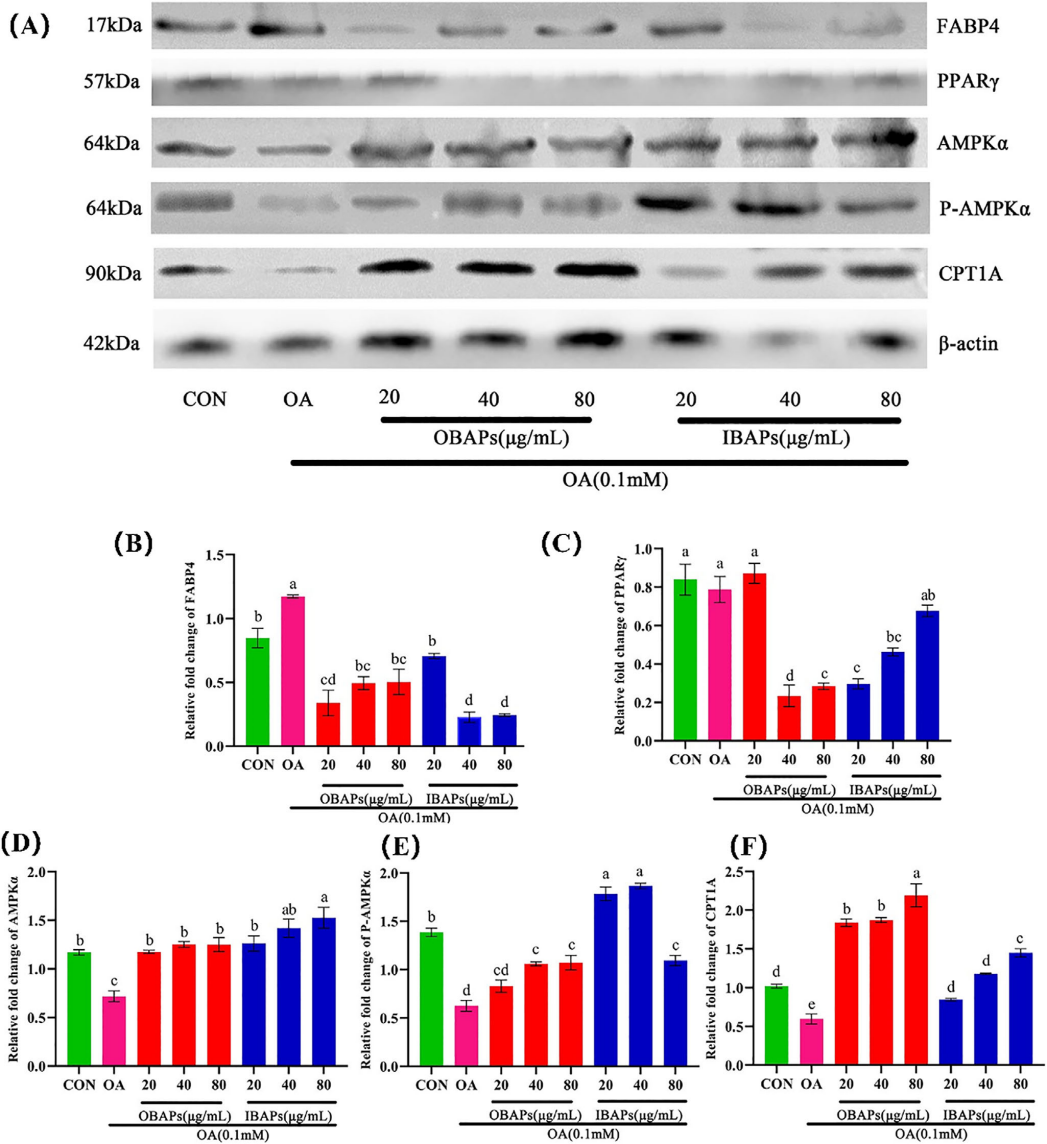
Western blot analysis of protein expression in HepG2 cells. (A) Representative blots of FABP4, PPARγ, AMPKα, P‐AMPKα, and CPT1A with *β*‐actin as the loading control. (B–F) Relative protein expression levels normalized to β‐actin. All data are presented as mean ± SEM from three independent biological replicates and analyzed by one‐way ANOVA with Tukey's post‐hoc test; significant differences (*p < 0.05) are indicated with different letters (a–d).

## 4 Discussion

In this study, the analysis of substances in artichokes unveiled substantial levels of caffeoylquinic acid (CQA) in both types of APs, including chlorogenic acid and 1,4‐dicaffeoylquinic acid, which constitute 60%–80% of the total polyphenols in artichoke. The studies conducted by Liu et al. [21] have shown that CQA has the potential to induce the conversion of white adipose tissue to brown adipose tissue, thereby enhancing the body's thermogenic capacity and addressing conditions associated with obesity. Additionally, findings suggest that chlorogenic acid plays a pivotal role in modulating lipid metabolism in genetic and metabolism‐related disorders, offering functional benefits for conditions such as hepatic steatosis, high cholesterol, cardiovascular diseases, diabetes, and obesity. Notably, chlorogenic acid has demonstrated a protective effect against liver damage induced by chemicals or lipopolysaccharides [22]. However, as the extracts are complex mixtures, the contribution of individual compounds, as well as potential synergistic effects among different polyphenolic constituents, cannot be determined from the present data and awaits future investigation using purified compounds. Notably, a qualitative comparison of the phytochemical profiles and biological activities between OBAPs and IBAPs revealed a consistent trend: the extract with a higher relative abundance of phenolic acids (IBAPs) exhibited slightly stronger lipid‐lowering activity than OBAPs, which contained higher proportions of flavonoids. However, quantitative correlation analyses between individual compounds and specific activity parameters were not performed in this study due to the semi‐quantitative nature of our phytochemical data. Future studies employing absolute quantification, fractionation, reconstitution approaches, or purified compound combinations will be essential to establish robust compositional‐activity relationships and to dissect the relative contributions of individual constituents and their potential synergistic or antagonistic interactions.

Comparing our findings with previous studies, several consistencies and novel observations emerge. Previous investigations have demonstrated that artichoke leaf extracts reduce plasma cholesterol and hepatic lipid accumulation in both clinical trials and animal models [7, 13], consistent with our observation that artichoke bract polyphenols lower intracellular TG and TC levels in HepG2 cells. The role of chlorogenic acid and other caffeoylquinic acids in modulating lipid metabolism has been well documented [21, 22], supporting our finding that APs rich in these compounds are associated with reduced lipid accumulation. Our results are also in line with studies showing that polyphenolic extracts from various plant sources activate the AMPK pathway in HepG2 cells [15, 18], reinforcing the notion that AMPK activation may represent a common mechanism underlying the lipid‐lowering effects of dietary polyphenols [23].

However, our study extends these previous observations in several important ways. First, unlike most prior work that focused on single polyphenolic compounds or leaf extracts, we systematically compared two distinct anatomical parts of the artichoke (outer versus inner bracts) and linked their compositional differences to differential biological activity. Second, while many previous studies used absolute quantification of selected compounds, our semi‐quantitative approach across all detected polyphenols allowed a broader comparison of the overall phytochemical profiles between OBAPs and IBAPs. Third, we observed that the higher relative abundance of phenolic acids in IBAPs correlated with slightly stronger activity, whereas OBAPs—which contain higher proportions of flavonoids—still retained substantial lipid‐lowering efficacy, suggesting that multiple polyphenolic classes may contribute to the observed effects. These findings not only confirm the anti‐steatotic potential of artichoke polyphenols but also highlight the valorization potential of artichoke bracts, including the typically discarded outer bracts, as functional ingredients.

Fatty Acid Binding Protein 4 (FABP4), a member of the fatty acid binding protein family, plays a fundamental role in metabolic processes. It facilitates the uptake and transportation of fatty acids within adipocytes, and is involved in both the oxidation and synthesis of fatty acids [24]. Consequently, an increase in FABP4 expression could potentially result in metabolic irregularities and the excessive accumulation of fatty acids [25]. This study highlights that the supplementation of APs can significantly diminish the expression of FABP4, particularly the IBAPs. Peroxisome proliferator‐activated receptor gamma (PPARγ) is a nuclear receptor that regulates ligand‐activated control over glucose and lipid metabolism, vascular endothelial function, and inflammation [26]. Furthermore, within the PPARγ signaling pathway, FABP4 resides upstream of PPARγ, and its upregulation can enhance the expression of PPARγ [24]. The activation and overexpression of PPARγ foster the differentiation of adipocytes, modulate the expression of downstream genes implicated in sustaining the mature adipocyte phenotype, and engage in the glucose‐lipid metabolism of adipocytes [27]. PPARγ stands out as a paramount transcription factor in the differentiation of precursor adipocytes into adipocytes, while also facilitating the transformation of myocytes into adipocytes [28]. By supplementing with APs, a potential decrease in PPARγ levels may impede adipocyte formation, which may contribute to the reduction in intracellular lipid accumulation. However, as no experiments using PPARγ agonists, antagonists, or gene silencing approaches were performed, the causal role of PPARγ in the antisteatotic effects of APs cannot be determined from the present data. Future studies employing such functional interventions are warranted to confirm whether PPARγ signaling is indeed required for the observed lipid‐lowering effects.

Adenosine 5'‐monophosphate‐activated protein kinase (AMPK), composed of a catalytic subunit (α) and two regulatory subunits (*β* and γ), wherein the α subunit (AMPKα) serves as a crucial sensor intricately linked to cellular energy metabolism [29]. Recent studies have highlighted that polyphenols can modulate AMPK and related signaling pathways, contributing to their beneficial metabolic effects [23]. Compared to the OA group, supplementation of artichoke polyphenols led to a noticeable increase in both AMPKα and P‐AMPKα expression, suggesting that artichoke polyphenols are associated with the activation of the AMPK signaling pathway. With increasing concentrations of artichoke polyphenols, there was a rising trend in the expression levels of AMPKα and P‐AMPKα. However, at an IBAPs concentration of 80 µg/mL, the expression of P‐AMPKα abruptly decreased, necessitating further investigation into the underlying reasons. Fatty acid synthase (FAS) furnishes triglycerides with non‐esterified fatty acid substrates, leading to escalated fatty acid synthesis and TG accumulation [30]. Phosphorylation of AMPKα is associated with increased expression of carnitine palmitoyl transferase I (CPT1A), a crucial rate‐limiting enzyme in fatty acid oxidation (FAO) that governs FAO and facilitates environmental adaptation, encompassing both health and disease states, including cancer [31]. Supplementation of artichoke polyphenols was associated with increased phosphorylation of AMPKα to P‐AMPKα, particularly with inner bract polyphenols, correlating with upregulation of CPT1A expression. This modulation of protein expression is consistent with a reduction in lipid accumulation, likely through decreased fatty acid synthesis and increased fatty acid oxidation, although direct measurements of fatty acid oxidation rates were not performed. Nevertheless, as no pharmacological AMPK inhibitors or gene knockdown experiments were performed, the causal relationship between AMPK activation and the antisteatotic effects of APs cannot be firmly established from the present data. The observed correlations between APs treatment, AMPK phosphorylation, and downstream lipid metabolism regulators should therefore be interpreted as hypothesis‐generating rather than definitive mechanistic proof. Future studies employing AMPK inhibitors (e.g., Compound C) or siRNA‐mediated AMPK knockdown will be required to confirm whether AMPK activation is indeed causally required for the lipid‐lowering effects of artichoke polyphenols.

Beyond the concentration discrepancy, the translational relevance of our findings is further constrained by the complex bioavailability and metabolism of artichoke polyphenols in humans. Caffeoylquinic acids, including chlorogenic acid and 1,4‐dicaffeoylquinic acid, are known to undergo extensive metabolism in the gastrointestinal tract and liver. Upon oral consumption, these compounds are subjected to hydrolysis by esterases in the intestine, metabolism by gut microbiota, and conjugation reactions (e.g., glucuronidation, sulfation, methylation) in the intestinal epithelium and liver. Consequently, the concentration of intact parent compounds reaching the systemic circulation is typically low, with circulating metabolites rather than the parent forms being the predominant species in plasma. Additionally, individual variations in gut microbiota composition and metabolic enzyme activities can substantially influence the bioavailability and bioactivity of dietary polyphenols. Thus, the APs concentrations tested in this study are not directly translatable to human physiological conditions. Nevertheless, our findings provide a mechanistic basis for understanding the potential effects of artichoke polyphenols at the cellular level and generate testable hypotheses for future investigations using appropriate in vivo models. Direct in vivo validation using established diet‐induced obesity or non‐alcoholic fatty liver disease animal models will be a critical next step to confirm the efficacy and safety of APs in a physiologically relevant context. Such studies should also evaluate dosage regimens, routes of administration, and the relationship between circulating metabolites and biological activity to better inform future translational applications.

The distinct phytochemical profiles between outer and inner bract polyphenols, particularly the enrichment of phenolic acids in IBAPs, may have practical implications for the valorization of artichoke by‐products. While IBAPs displayed slightly stronger activity, the substantial phenolic acid content in OBAPs suggests that even the discarded outer bracts hold promise as a functional ingredient for managing lipid‐related metabolic conditions, pending further in vivo validation.

It is important to note that OBAPs and IBAPs were tested at equal total phenolic concentrations (as determined by the Folin‐Ciocalteu method), rather than equal extract mass. Therefore, the observed differences in biological activity between the two extracts cannot be attributed to differences in overall phenolic content. Instead, these differences likely reflect variations in the compositional profiles of the two extracts, particularly the higher relative abundance of phenolic acids in IBAPs compared with the higher flavonoid content in OBAPs. While this qualitative observation is consistent with the hypothesis that phenolic acids contribute more substantially to the lipid‐lowering effects, we acknowledge that a formal statistical attribution of the observed activity differences to specific compositional variables would require multivariate correlation analyses with a larger number of extract batches or fractions. Such analyses were beyond the scope of the present study but represent a valuable direction for future investigations.

We acknowledge several limitations of the present study. First, the purity of the APs extracts was determined solely by the Folin‐Ciocalteu method, which quantifies total phenolic content but does not provide information on the non‐phenolic fraction. The remaining mass of the extracts (approximately 17%–24%) may include residual sugars, proteins, or other polar compounds that were not characterized. Future work employing comprehensive analytical approaches would be valuable to fully characterize these constituents.

Second, our phytochemical characterization is semi‐quantitative and based on tentative identification via HPLC‐MS/MS without authentic standards; future studies using authenticated standards are needed for definitive quantification.

Third, the APs concentrations used in this study (20–80 µg/mL) are higher than the plasma concentrations typically achievable through dietary consumption, which is a common limitation of in vitro studies using crude plant extracts. Future pharmacokinetic and in vivo studies are warranted to determine physiologically relevant concentrations and to validate the translational potential of these findings.

Fourth, while the CCK‐8 assay confirmed cell viability at the selected APs concentrations, additional assays evaluating apoptosis, membrane integrity, oxidative stress, or inflammatory mediators were not performed; such assessments would be valuable to confirm that the observed lipid reduction is not secondary to subtle cellular damage or stress‐related mechanisms.

Fifth, while our Western blot data demonstrate changes in protein expression levels, we did not perform corresponding mRNA expression analyses. Therefore, whether the observed protein changes are regulated at the transcriptional or post‐transcriptional level remains unknown.

Sixth, several key regulators of lipid metabolism—including ACC/p‐ACC, SREBP‐1c, CD36, DGAT, ATGL, and HSL—were not examined, and we did not perform direct functional assays to confirm enhanced mitochondrial fatty acid oxidation (e.g., OCR, ATP production, or β‐oxidation activity). Our conclusions regarding these pathways are therefore inferences based on protein expression changes.

Seventh, while the APs extracts were stored as lyophilized powders at −20°C in the dark and freshly dissolved before each experiment, the stability of individual polyphenolic compounds during cell culture incubation was not systematically evaluated. Polyphenols can be susceptible to degradation under cell culture conditions, which may influence the actual concentrations of bioactive compounds reaching the cells.

Eighth, additional complementary assays—such as BODIPY staining, intracellular free fatty acid quantification, untargeted metabolomic or lipidomic analyses—were not performed. Furthermore, the investigators performing image acquisition and quantification were not blinded to the treatment groups. These approaches would provide further validation and objectivity in future studies.

## 5 Conclusion

This study demonstrates that artichoke bract polyphenols (APs), particularly those from inner bracts (IBAPs), alleviate oleic acid‐induced hepatic lipid accumulation in HepG2 cells. Semi‐quantitative HPLC‐MS/MS analysis revealed that APs are predominantly composed of phenolic acids, with 1,4‐dicaffeoylquinic acid and chlorogenic acid comprising 60%–80% of the total polyphenolic content. Functional assays confirmed that APs dose‐dependently reduced intracellular lipid droplets, triglyceride, and total cholesterol levels without compromising cell viability.

Mechanistically, APs modulated the PPARγ and AMPK signaling pathways: they downregulated the expression of FABP4, and PPARγ, while increasing AMPKα phosphorylation and CPT1A expression. These molecular changes are consistent with enhanced fatty acid oxidation (as inferred from CPT1A upregulation) and reduced de novo lipogenesis. Notably, IBAPs, which were characterized by higher relative abundance of caffeoylquinic acids, exhibited slightly stronger lipid‐lowering activity than OBAPs in certain assays (e.g., reducing lipid accumulation and TC content); however, the differences were modest and require further confirmation.

These in vitro findings suggest that artichoke bract polyphenols, including those from the typically discarded outer bracts, hold promise as natural functional agents that warrant further investigation for managing obesity‐related metabolic disorders. However, the present study has several limitations—including the tentative nature of compound identification, the use of a single transformed hepatic cell line, and the lack of direct enzyme activity or causal mechanistic validation—which are comprehensively discussed in the Discussion section (Section 4). Future research employing in vivo models (e.g., high‐fat diet‐induced obese mice) and functional mechanistic approaches (e.g., AMPK inhibitors, gene silencing, or purified compound testing), along with pharmacokinetic and bioavailability assessments, will be essential to confirm the translational potential of APs and to elucidate the precise contributions of individual phenolic constituents.

## Author Contributions

**Wenyang Tao**: constructed high‐fat cell models, detected intracellular lipid accumulation, triglyceride and total cholesterol levels; performed preliminary data analysis; drafted the original manuscript. **Chenchen Yang**: assisted with cell functional experiments, sample pretreatment, and partial physiological index detection; sorted and proofread raw experimental data. **Quanqin Tan**: extracted, separated, and purified artichoke polyphenols; performed HPLC‐MS analysis and identified phenolic acid components, including 1,4‐dicaffeoylquinic acid. **Wanyi Zhou**: conducted in‐depth data statistical analysis and visualization; sorted data tables and assisted with result interpretation. **Jianrong Xing**: prepared experimental reagents and cell culture materials; assisted with experimental condition optimization and routine tests. **Jingrui Li**: participated in PPAR and AMPK pathway mechanism analysis; assisted with protein expression and signal pathway result analysis. **Mengfan Luo**: collected literature, standardized references; revised manuscript language, logic and format. **Ying Yang**: designed the overall research scheme, provided funding and platform support; supervised the whole experiment, data verification and manuscript revision; finalized the manuscript and handled academic correspondence.

## Funding

This work was supported by the Key Research and Development Program of Zhejiang Province, China (Grant No. 2020C02034).

## Conflicts of Interest

The authors declare no conflicts of interest.

## Data Availability

The data that support the findings of this study are available from the corresponding author upon reasonable request.
